# Polymorphisms in Base Excision Repair Genes and Association with Multiple Sclerosis in a Pilot Study on a Central European Population

**DOI:** 10.3390/ijms26146612

**Published:** 2025-07-10

**Authors:** Beata Filipek, Anna Macieja, Aleksandra Binda, Elzbieta Miller, Mariola Swiderek-Matysiak, Mariusz Stasiolek, Maksymilian Stela, Ireneusz Majsterek, Tomasz Poplawski

**Affiliations:** 1 Department of Microbiology and Pharmaceutical Biochemistry, Medical University of Lodz, Mazowiecka 5, 92-215 Lodz, Poland; beata.filipek@umed.lodz.pl (B.F.); anna.macieja@umed.lodz.pl (A.M.); 2 Department of Neurology, Medical University of Lodz, Kopcinskiego 22, 90-153 Lodz, Poland; mariola.swiderek-matysiak@umed.lodz.pl (M.S.-M.); mariusz.stasiolek@umed.lodz.pl (M.S.); 3 Department of Clinical Chemistry and Biochemistry, Medical University of Lodz, Mazowiecka 5, 92-215 Lodz, Poland; aleksandra.binda@umed.lodz.pl; 4 Department of Neurological Rehabilitation, Medical University of Lodz, Milionowa 14, 93-113 Lodz, Poland; elzbieta.dorota.miller@umed.lodz.pl (E.M.); ireneusz.majsterek@umed.lodz.pl (I.M.); 5 Biohazard Prevention Centre, University of Lodz, Pomorska 141/143, 90-236 Lodz, Poland; maksymilian.stela@biol.uni.lodz.pl

**Keywords:** multiple sclerosis, base excision repair, gene polymorphisms

## Abstract

Multiple sclerosis (MS) is a chronic inflammatory disease of the central nervous system characterized by demyelination and neurodegeneration. While its etiology remains unclear, both genetic and environmental factors, including oxidative stress, have been implicated in the development of the disease. The base excision repair (BER) pathway plays a critical role in repairing oxidative DNA damage. This study investigated the association between polymorphisms in BER-related genes and MS susceptibility in a Central European population. Ten SNPs across seven BER genes were genotyped in 102 patients with MS and 118 healthy controls. Six SNPs were significantly associated with MS. Increased risk was observed for rs25478 in XRCC1 (OR = 2.37, 95% CI: 1.44–3.91, *p* < 0.0001), rs3087404 in SMUG1 (OR = 2.80, 95% CI: 1.49–5.26, *p* = 0.0012), and rs3219493 in MUTYH (OR = 2.23, 95% CI: 1.35–3.67, *p* = 0.0018). Conversely, reduced risk was associated with rs2307293 in MBD4 (OR = 0.42, 95% CI: 0.23–0.78, *p* = 0.006), rs3219489 in MUTYH (OR = 0.55, 95% CI: 0.31–0.97, *p* = 0.038), and rs4135054 in TDG (OR = 0.52, 95% CI: 0.29–0.94, *p* = 0.031). Haplotype analysis was performed for SNPs in strong linkage disequilibrium. Only rs3219489 and rs3219472 within the MUTYH gene showed strong LD (r^2^ = 0.90), justifying haplotype-based analysis. Among four inferred haplotypes, the rare G–C haplotype was significantly associated with reduced MS risk (Score = −2.10, *p* = 0.035), suggesting a protective effect of this allele combination. Other SNPs not in LD were analyzed using a multivariable logistic regression model. Significant associations with decreased MS risk were found for rs1052133 in OGG1 (OR = 0.57, *p* = 0.043), rs2307293 in MBD4 (OR = 0.16, *p* = 0.010), and rs4135054 in TDG (OR = 0.38, *p* < 0.001), while rs3087404 in SMUG1 increased MS risk (OR = 1.98, *p* = 0.013). These results suggest that genetic variation in BER genes, including both single SNP effects and haplotypes, contributes to MS susceptibility. Further studies are warranted to explore the functional consequences of these variants and validate findings in larger, independent cohorts.

## 1. Introduction

Multiple sclerosis (MS) is a chronic inflammatory disease of the central nervous system (CNS), characterized by demyelination, neurodegeneration, and various neurological symptoms. Relapsing–remitting MS (RRMS) accounts for approximately 80–85% of initial MS diagnoses, while primary progressive MS (PPMS) and secondary progressive MS (SPMS) together represent the remaining 15–20% [1]. Magnetic resonance imaging (MRI) plays a key role in diagnosing and monitoring MS, allowing the detection of characteristic white matter lesions in the periventricular, juxtacortical, infratentorial, and spinal cord regions. Gadolinium-enhancing lesions indicate active inflammation, whereas T1-hypointense and T2-hyperintense lesions show tissue damage and demyelination. MRI also aids in assessing lesion dissemination in space and time, as required by the McDonald criteria. Patients with MS may experience a wide range of symptoms, such as visual impairment, motor dysfunction, ataxia, sensory disturbances, and cognitive decline. Diagnosis is based on clinical presentation and radiological criteria [2].

MS is one of the leading causes of disability among young adults, affecting approximately 2.9 million people worldwide. In Poland alone, there are over 51,000 patients diagnosed with MS, with a prevalence of 134 per 100,000 people [2,3]. The peak onset of MS typically occurs between the ages of 20 and 40 [1,2].

Although the exact cause of MS remains unclear, it is widely accepted that a combination of genetic and environmental factors influences disease susceptibility. In addition to known triggers such as Epstein–Barr virus infection, smoking, low vitamin D levels, and environmental toxins, oxidative stress has become an essential factor in both the development and progression of MS [4,5,6,7]. Elevated levels of oxidative stress markers and lower antioxidant capacity have been observed in patients with MS, indicating an imbalance between the production of reactive oxygen species (ROS) and antioxidant defenses [8,9]. This imbalance is believed to contribute to neurodegeneration and inflammation within the CNS. Major contributors to oxidative stress in MS include mitochondrial dysfunction, neuroinflammation, iron ion dysregulation, and immune system activation [10,11,12].

In response to oxidative DNA damage, cells activate base excision repair (BER), the primary DNA repair pathway that fixes oxidative lesions. BER begins with DNA glycosylases, which identify and remove damaged bases, creating apurinic/apyrimidinic (AP) sites. These sites are then processed by AP endonuclease 1 (APE1), DNA polymerase β, and DNA ligase III to restore DNA integrity [13,14]. Among the key glycosylases involved—hOGG1, hNTH1, hNEIL1, hNEIL2, and hMYH—each has specific substrate preferences and repairs different oxidative lesions, such as 8-oxo-7,8-dihydroguanine, thymine glycol, and hydantoin derivatives [15,16,17]. If left unrepaired, oxidative DNA lesions can block transcription and replication, leading to genomic instability, faster cellular aging, and impaired normal CNS function.

Genetic variation in BER genes may affect the efficiency of DNA repair mechanisms, thereby influencing susceptibility to damage caused by oxidative stress. Single-nucleotide polymorphisms (SNPs) in BER-related genes have been linked to various inflammatory and neurodegenerative conditions.

While genome-wide association studies (GWASs) have successfully identified over 200 genetic loci linked to MS susceptibility [18,19], they are often limited in their ability to discover common variants with small effect sizes. They may not fully detect functional variants within key biological pathways [20]. Additionally, GWAS signals often span broad linkage disequilibrium (LD) blocks, making it difficult to identify causal variants or understand their mechanistic roles [21]. Rare or moderately common variants with larger biological effects might be missed due to limited statistical power or inadequate tagging [22].

In contrast, candidate gene studies enable hypothesis-driven analysis of variants with known or suspected functional roles, such as those in the base excision repair (BER) pathway, which is crucial for counteracting oxidative stress, a recognized contributor to MS pathophysiology [4,5,6,7]. By focusing on the BER pathway, our study aims to complement GWASs by examining functionally relevant SNPs that may affect individual susceptibility to oxidative stress and neuroinflammation in MS. Therefore, this study aimed to evaluate the presence of selected SNPs within BER genes in patients with MS. We chose 10 SNPs (rs25478, rs1052133, rs246079, rs151095402, rs2307293, rs3219472, rs3219489, rs3219493, rs4135054, and rs3087404). The selection of these SNPs was based on their previous association with various diseases characterized by chronic inflammation, oxidative stress, and DNA repair deficiencies, including cancer, age-related macular degeneration, and autoimmune disorders [23,24,25,26]. Although no prior studies have specifically examined these polymorphisms in MS, their functional annotation and relevance to BER mechanisms justified their inclusion in this hypothesis-driven pilot study.

## 2. Results

### 2.1. Characteristics of the Study Population

The demographic characteristics of the study groups are summarized below. The mean age of patients with MS was 42.2 ± 13.1 years, while the mean age of controls was 38.4 ± 16.7 years. The age distribution difference between the groups was not statistically significant (*p* = 0.07, Student’s *t*-test). Regarding sex distribution, the MS group consisted of 62 females and 40 males, while the control group consisted of 70 females and 48 males. There was no significant difference in sex distribution between the groups (*p* = 0.89, chi-squared test). The summary of the selected demographic and clinical characteristics of MS cases is presented in Table 1. This study included 102 patients with MS (mean age ± SD: 42.2 ± 13.12)—62 women (mean age ± SD: 42.29 ± 13.02) and 40 men (mean age ± SD: 42.05 ± 13.43)—from the Department of Neurology and the Department of Neurological Rehabilitation at the Medical University of Lodz. All participants met McDonald’s criteria for MS 2017 [1]. Nearly all patients had relapsing–remitting MS (RRMS). Disease severity was assessed using the Expanded Disability Status Scale (EDSS), yielding a score of 3.44 (±1.87). Most patients were treated with ocrelizumab (*n* = 30) or natalizumab (*n* = 22). Other treatments included dimethyl fumarate (*n* = 14), fingolimod (*n* = 2), glatiramer acetate (*n* = 6), interferon beta-1a (*n* = 3), ofatumumab (*n* = 12), and siponimod (*n* = 3). Ten patients were not on disease-modifying therapy (DMT) at the time of sample collection; these individuals were either treatment-naïve or recently diagnosed and awaiting therapy. Patients with MS did not have any additional inflammatory diseases or cancer.

### 2.2. The Hardy–Weinberg Equilibrium Analysis

Test results for the Hardy–Weinberg (HW) principle are presented in Table 2. Hardy–Weinberg equilibrium (HWE) was evaluated in the control group for all SNPs. After applying the Bonferroni correction for multiple testing (adjusted significance threshold: *p* < 0.005), none of the SNPs showed a significant deviation from HWE, indicating a reliable genotype distribution and no evidence of genotyping error or population stratification in the controls.

### 2.3. Analysis of the Relationship Between the Occurrence of MS and the Studied Polymorphic Variants of BER Genes

This assessment was conducted through association studies, which are population-based investigations designed to determine whether a specific gene allele is more prevalent in individuals with MS compared to healthy individuals. For each polymorphism, the frequency of each genotype was provided concerning whether MS was present or absent (Table 3).

**Figure 1:**
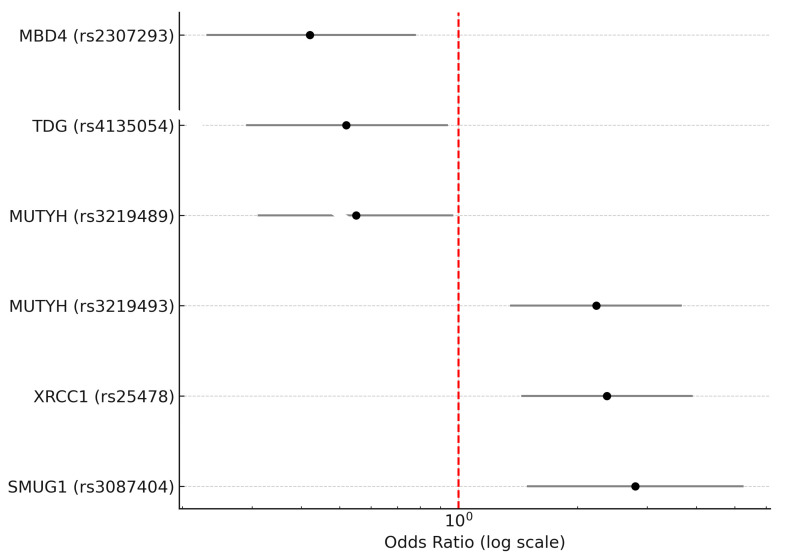
Forest plot illustrating odds ratios (ORs) with 95% confidence intervals (CIs) for six single-nucleotide polymorphisms (SNPs) in base excision repair (BER) genes that showed statistically significant associations with multiple sclerosis (MS). Three SNPs—rs25478 in XRCC1, rs3087404 in SMUG1, and rs3219493 in MUTYH—were associated with an increased risk of MS (OR > 1), while three others—rs2307293 in MBD4, rs3219489 in MUTYH, and rs4135054 in TDG—were associated with a decreased risk of MS (OR < 1). The horizontal bars represent 95% confidence intervals. The red vertical dashed line indicates an odds ratio of 1.0 (no association). Odds ratios are plotted on a logarithmic scale. Only SNPs with *p*-values < 0.05 after Bonferroni correction are included.

Four genetic models were analyzed to understand their association with MS: codominant, dominant, recessive, and superdominant. When examined under the codominant, dominant, and recessive models, this study revealed a significant correlation between MS and the rs4135054 polymorphism in the TDG gene. Similarly, the rs2307293 polymorphism in the MBD4 gene showed strong associations with MS across codominant, dominant, and overdominant models. Furthermore, the rs3219489 polymorphism in the MUTYH gene was linked to MS in both codominant and overdominant frameworks. Additionally, our findings indicated a noteworthy association between MS and various genetic models of the rs3087404 polymorphism in the SMUG1 gene, supporting its potential role in MS development. Lastly, the rs25478 polymorphism in the XRCC1 gene exhibited significant correlations with MS in both codominant and recessive models. Detailed results for these associations are presented in Table 4, accompanied by a graphical summary in Figure 1.

Based on pairwise linkage disequilibrium (LD) analysis using r^2^ values, only two SNPs within the MUTYH gene (rs3219489 and rs3219472) showed strong LD (r^2^ = 0.90), justifying haplotype-based analysis for this gene (Table 5). Four haplotypes were identified, with the C–C haplotype being the most frequent (82.3%). The overall test for haplotype–phenotype association indicated a trend toward significance (χ^2^ = 6.63, df = 3, *p* = 0.085). Notably, the rare haplotype G–C was significantly associated with a decreased risk of MS (Score = −2.10, *p* = 0.035), suggesting a possible protective effect for this allele combination.

The remaining SNPs, including rs25478 (XRCC1), rs1052133 (OGG1), rs246079 (APE1), rs3087404 (SMUG1), rs151095402 (UNG), rs2307293 (MBD4), rs3219493 (MUTYH), and rs4135054 (TDG), exhibited weak or negligible LD (r^2^ < 0.05) and were thus analyzed using a multilocus logistic regression model (Table 6).

A multilocus logistic regression was conducted, including eight SNPs from BER-related genes located on different chromosomes or outside known LD blocks. The model demonstrated a good fit (AIC = 269.31; residual deviance = 251.31). Significant associations with reduced MS risk were observed for rs1052133 (OGG1, OR = 0.57, *p* = 0.043), rs2307293 (MBD4, OR = 0.16, *p* = 0.010), and rs4135054 (TDG, OR = 0.38, *p* < 0.001). Conversely, rs3087404 (SMUG1) was associated with an increased risk (OR = 1.98, *p* = 0.013). A trend was also noted for rs25478 (XRCC1, OR = 2.00, *p* = 0.068). Other variants, including rs3219493 (MUTYH), rs151095402 (UNG), and rs246079 (APE1), did not show significant associations.

To investigate potential epistatic interactions, three biologically plausible SNP × SNP combinations were tested using logistic regression models with multiplicative interaction terms: XRCC1 × OGG1 (rs25478 × rs1052133), SMUG1 × TDG (rs3087404 × rs4135054), and OGG1 × MBD4 (rs1052133 × rs2307293). The selection of these pairs was based on the biological functions of the proteins involved in the base excision repair (BER) pathway, which repairs oxidative DNA damage through a coordinated series of steps. The first pair tested was XRCC1 × OGG1 (rs25478 × rs1052133), where OGG1 is a DNA glycosylase that excises 8-oxoguanine, and XRCC1 serves as a scaffold protein interacting with DNA ligase III and other repair enzymes to coordinate repair. A possible functional interaction may influence the efficiency with which the repair complex forms after oxidative damage. The second pair, SMUG1 × TDG (rs3087404 × rs4135054), includes two glycosylases with overlapping substrates for uracil and modified pyrimidines. Their cooperative or compensatory activity could modulate the cellular response to deamination or misincorporation events, especially in active transcription regions. Finally, the OGG1 × MBD4 interaction (rs1052133 × rs2307293) was examined because MBD4 links glycosylase activity with epigenetic regulation and mismatch repair. Since OGG1 initiates the repair of oxidative base lesions, and MBD4 affects chromatin structure and G:T mismatch processing at CpG sites, their combined variation may impact DNA repair in regulatory genomic regions. None of the interactions achieved statistical significance (*p* > 0.4 in all cases), and no synergistic effects were observed. The main effects of the individual SNPs remained consistent across models, supporting an additive rather than interactive mode of association in the current cohort.

Based on the power analysis performed with QUANTO, this study’s sample size of 102 patients with MS and 118 controls was enough to achieve 80% power to detect an odds ratio of 1.8 under a log-additive model, assuming minor allele frequencies between 0.1 and 0.4. This confirmed that the sample was sufficiently powered to detect moderate genetic effects associated with the selected SNPs. Therefore, this study fulfills the statistical criteria for reliable interpretation of the association results.

## 3. Discussion

The genetic background of multiple sclerosis (MS) involves a complex interaction of genetic, environmental, and random factors, with significant input from both common and rare genetic variants. The most consistent genetic risk factor for MS is the HLA-DRB1*15:01 allele, located within the major histocompatibility complex (MHC) on chromosome 6p21, which has been shown to increase the risk of the disease [27,28,29] significantly. Genome-wide association studies (GWASs) have also identified several non-HLA genetic variants linked to MS, including those in the interleukin-7 receptor (IL7RA), interleukin-2 receptor (IL2RA), CD58, and CLEC16A genes, indicating a shared genetic foundation with other autoimmune diseases [30]. Despite these discoveries, the genetic architecture of MS is polygenic, with many genes each exerting minor effects, and the overall genetic influence on MS risk is considered modest, as shown by the low concordance rates in monozygotic twins [31,32]. Research has also underscored the importance of gene–environment interactions, such as those involving Epstein–Barr virus (EBV) and vitamin D, which can influence the risk associated with genetic factors [28]. Furthermore, genetic susceptibility to MS varies among populations, with certain genetic associations being more common in specific ethnic groups, as demonstrated by studies from the Volga-Ural region of Russia [33]. The absence of significant linkage findings in genome scans further highlights the complexity of MS genetics. This suggests that MS is influenced by multiple loci with minor effects, rather than a few genes of major importance [34]. While considerable progress has been made in identifying genetic factors related to MS, the exact mechanisms underlying disease development are still not fully understood, requiring further investigation into gene–gene and gene–environment interactions [35,36].

This study appears to be the first to identify an association between MS occurrence and polymorphisms of the BER genes. This discovery opens new pathways for understanding the role of DNA repair mechanisms in MS development. It highlights the importance of examining how variations in these genes may influence an individual’s susceptibility to the disease. Our evidence indicates that rs2307293/MBD4, rs3219489/MUTYH, and rs4135054/TDG SNPs have a protective effect against MS, while rs3087404/SMUG1 and rs25478/XRCC1 have the opposite effect.

Multimarker and interaction-based analyses further enhanced our understanding of how BER gene polymorphisms influence the risk of MS. Haplotype analysis showed strong linkage disequilibrium (r^2^ = 0.90) between rs3219489 and rs3219472 in MUTYH, enabling biologically and statistically justified haplotype modeling. Of the four reconstructed haplotypes, the rare G–C combination was significantly linked to a lower risk of MS (*p* = 0.035), indicating that specific allelic combinations within MUTYH may offer protective effects beyond individual SNP associations. Meanwhile, a multivariable logistic regression model was created for SNPs not in LD. This analysis confirmed the independent protective effects of rs1052133 (OGG1), rs2307293 (MBD4), and rs4135054 (TDG), as well as the increased risk associated with rs3087404 (SMUG1), while accounting for potential confounding factors among unlinked loci. Interestingly, rs25478 (XRCC1) showed only a trend toward significance in the adjusted model, implying that other loci might partly influence its risk association. Lastly, we tested for potential epistatic interactions between biologically plausible SNP pairs within the BER pathway. None of the interaction terms (XRCC1 × OGG1, SMUG1 × TDG, OGG1 × MBD4) achieved statistical significance (*p* > 0.4), suggesting an additive rather than synergistic effect of these loci in our cohort. These multilocus results highlight the complex, polygenic role of BER gene variation in MS susceptibility, suggesting that integrated analytical strategies are crucial for future research.

SMUG1 mainly removes uracil and oxidized pyrimidines from DNA [37,38]. It features a unique helical wedge structure that facilitates damage recognition during DNA repair [37]. SMUG1’s activity is not limited to DNA repair; it also plays essential roles in RNA processing, including in the RNA component of telomerase, showing its multifunctionality beyond BER [37], which highlights its vital role in maintaining genomic stability and cellular function.

The rs3087404/SMUG1 SNP is a single-nucleotide polymorphism located in the promoter region of the SMUG1 gene, which encodes the single-strand-selective monofunctional uracil–DNA glycosylase 1. This polymorphism involves an A-to-G substitution at position 31 in the promoter region (c.-31A>G) and may influence the expression of the SMUG1 gene due to its location. The rs3087404/SMUG1 SNP has been linked to various diseases, including an increased risk of cervical intraepithelial neoplasia III (CIN III) [37] and cervical squamous cell carcinoma (CSCC) in HR-HPV-positive individuals [38], as well as a potential role in the development of age-related macular degeneration (AMD) [39] and the onset and recurrence of depressive disorder [40]. This SNP has been studied in conjunction with other polymorphisms, such as the rs2337395/UNG polymorphism [41]. These connections suggest that rs3087404/SMUG1 could serve as a genetic marker for disease risk or treatment response in certain conditions, indicating its possible clinical significance. The second SNP (rs25478/XRCC1) is much better characterized than the first. rs25487/XRCC1 is situated in exon 10 of the XRCC1 gene, which plays an essential role in BER [42]. This SNP causes an amino acid change from arginine to glutamine at position 399, potentially affecting DNA repair function [42]. The G allele encodes arginine, while the A allele encodes glutamine, with a global minor allele frequency of 0.2631 [42]. rs25487/XRCC1 has been linked to increased risks for various diseases, mainly cancers, including breast cancer in American populations and lung cancer in susceptible Chinese populations [43]. It has also been associated with a higher risk of hepatocellular carcinoma, though no significant link was found with nasopharyngeal carcinoma characteristics [44,45]. Regarding radiotherapy response, this SNP correlates with a higher risk of mild treatment response and poor overall survival in patients with cancer undergoing radiotherapy, with carriers more prone to side effects, especially in head and neck cancers [46]. Beyond cancer, the CC genotype and C allele of rs25487 are significantly associated with susceptibility to HIV-1 infection and may influence nicotine dependence [47]. Functionally, rs25487 might decrease the affinity between XRCC1 and the DNA repair complex, possibly impairing repair capacity [41]. Notably, the frequency of alleles and genotype distributions of rs25487 differ significantly among populations, with the Central Saudi population showing a distinctive pattern compared to others [48]. Given its associations with various diseases and treatment outcomes, rs25487 has potential as a clinical marker for cancer risk assessment and radiotherapy response, which could aid in personalizing treatment strategies. However, the current findings suggest that the clinical role of this polymorphism may extend beyond cancer, especially in diseases where BER and related disorders are relevant to disease development.

Of the three analyzed polymorphisms that reduce the risk of MS, rs3219489/MUTYH appears to be the most thoroughly described in a clinical context. This SNP is located in the MUTYH gene on chromosome 1 and results in an amino acid change from glutamine (Q) to histidine (H) at position 338 (Q338H), potentially affecting the DNA glycosylase function involved in the repair of oxidative DNA damage [49]. The G allele encodes glutamine, while the C allele encodes histidine, with a global minor allele frequency of 0.3186. rs3219489/MUTYH has been associated with various cancer risks, including a decreased risk of colorectal cancer (CRC) for the CG + GG genotype in some studies. However, a large meta-analysis found no significant association with CRC risk [25]. It has also been linked to a decreased risk of age-related macular degeneration (AMD) [50]. These observations are consistent with our own regarding the direction of the effect. Notably, the allele frequencies of rs3219489 vary significantly among different populations, with the C allele (encoding histidine) being more common in Japanese and Chinese populations than in Caucasians. Functionally, the C allele may be associated with a partially inactive base excision repair function, potentially impacting the DNA repair capacity of the MUTYH enzyme [51]. In terms of clinical implications, rs3219489/MUTYH may serve as a genetic marker for cancer risk assessment, especially in specific populations, and could influence the effectiveness of radiochemotherapy in patients with non-small-cell lung cancer (NSCLC) [51]. However, it is essential to note that while numerous studies have shown associations between rs3219489/MUTYH and various conditions, results can vary across different populations and cancer types, underscoring the need for further research to understand its role in disease susceptibility and treatment response fully.

A complex interplay of genetic, environmental, and random factors shapes the genetic landscape of MS. Although the HLA-DRB1*15:01 allele remains the most consistently identified genetic risk factor, recent studies have highlighted the potential role of DNA repair genes, especially those involved in BER, in MS susceptibility. This study’s novel findings suggest that polymorphisms in BER genes may influence MS risk. These associations open new avenues for understanding how DNA repair mechanisms contribute to MS development. Due to its well-understood clinical context in other conditions, the rs3219489/MUTYH SNP primarily shows promise as a potential genetic marker for MS risk assessment. However, it is essential to remember that MS has a polygenic genetic architecture, with many genes exerting minor effects. The overall genetic contribution to MS risk remains modest, as shown by low concordance rates in identical twins. Additionally, gene–environment interactions, such as those involving the Epstein–Barr virus and vitamin D, may influence the risk associated with genetic factors. While these findings mark a significant step forward in our understanding of MS genetics, further research is needed to clarify how these BER gene polymorphisms contribute to MS development. Future studies should also investigate potential gene–gene and gene–environment interactions related to MS. Our pilot study involved an ethnically homogeneous Polish population; therefore, validating these associations in diverse populations is necessary, considering the known genetic differences in MS susceptibility among various ethnic groups.

Although genome-wide association studies (GWASs) have successfully identified many common genetic variants linked to multiple sclerosis (MS), these loci generally have small effect sizes with odds ratios usually below 2, reflecting the complex polygenic nature of MS. GWASs tend to focus on common variants with relatively high minor allele frequencies but may miss rare or low-frequency variants that could have larger biological impacts. Additionally, GWAS signals often point to broad linkage disequilibrium regions, leaving uncertainty about the exact causal variants and their functional significance.

Candidate gene association studies, like ours, remain a valuable complementary approach for several reasons. First, they enable targeted, hypothesis-driven exploration of functionally relevant variants within biologically plausible pathways, such as the base excision repair (BER) pathway, which is involved in oxidative DNA damage, a key factor in MS pathophysiology. Second, focused studies can analyze variants that are not well-tagged or captured in GWAS arrays, including those with moderate minor allele frequencies or functional annotations indicating a direct effect on protein function or gene regulation. Third, candidate gene studies offer opportunities to examine gene–gene and gene–environment interactions with greater precision, which large-scale GWASs often cannot fully address because of multiple testing penalties and statistical limitations.

Although our sample size is smaller compared to GWAS cohorts, this study is adequately powered to detect moderate effect sizes within the selected SNPs, as confirmed by our power calculations. Our findings thus serve as an essential resource to validate and expand GWAS results at the pathway level, generate mechanistic hypotheses, and inform future large-scale replication and functional studies. This approach is especially valuable in ethnically homogeneous populations, like our Central European cohort, where population-specific genetic architectures may influence MS susceptibility.

Our study complements GWASs by offering a hypothesis-driven, mechanistically based analysis of BER gene polymorphisms that may influence MS susceptibility. While GWASs have been successful in identifying common variants with modest effects, candidate gene studies like ours clarify the biological relevance of specific variants within key pathways such as oxidative DNA repair. The associations we identified, particularly those involving XRCC1, SMUG1, TDG, MBD4, and MUTYH, underscore the potential functional significance of BER-related genetic variation. These findings could lay the groundwork for future research focused on functional validation, exploring gene–environment interactions, and developing BER-related polymorphisms as predictive biomarkers in MS.

While this study focused on genetic associations, quantitative MRI parameters—including demyelinating lesion volume, brain atrophy, active lesion burden, paramagnetic rim lesions, and central vein sign—are currently being analyzed in the same patient cohort and will be presented in an upcoming publication.

Please note that although most patients in our study had relapsing–remitting MS (RRMS), a small number presented with primary progressive (n = 3) and secondary progressive (n = 1) forms. These subtypes may have different genetic susceptibilities; however, given their limited representation (3.9%), their effect on the overall association results is probably minimal. Still, this clinical diversity should be recognized as a limitation, and future studies should include stratified analyses across MS phenotypes. Similarly, although age at disease onset was recorded, it was not included in the current statistical model and should be considered in future studies with larger, more diverse cohorts.

## 4. Materials and Methods

### 4.1. Characteristics of the Study Population

We included 102 patients with MS (62 women and 40 men; mean age 42.2 ± 13.12 years) selected from patients treated at the Department of Neurology and Department of Neurological Rehabilitation, Medical University of Lodz. The control group consisted of 118 subjects (70 women and 48 men; mean age 38.36 ± 16.7 years) without any diagnosed chronic inflammatory conditions. The Institutional Bioethics Committee of the Medical University of Lodz, Lodz, Poland (No RNN/235/20/KE), approved the study. All patients with MS fulfilled the McDonald’s criteria for MS 2017 [1].

### 4.2. DNA Isolation

Blood samples were collected from patients and controls into anticoagulant tubes (up to 9 mL). Following the manufacturer’s protocol, we isolated DNA using the GeneMatrix Blood DNA Purification Kit (EURx, Gdansk, Poland). After isolation, DNA samples were stored at −20 °C in a Tris/EDTA (TE) buffer (pH 8.0). DNA concentration and purity were measured spectrophotometrically by assessing absorbance at 260 and 280 nm on a Synergy HT spectrophotometer (BioTek, Hong Kong, China).

### 4.3. Determination of Single-Nucleotide Polymorphisms (SNPs)

The frequency of polymorphic variants of genes—X-Ray Repair Cross-Complementing 1 (XRCC1) (rs25478), 8-oxoguanine DNA glycosylase (OGG1) (rs1052133), uracil–DNA glycosylase (UNG) (rs246079 and rs151095402), Methyl-CpG-Binding Domain 4 (MBD4) (rs2307293), MutY DNA glycosylase (MUTYH) (rs3219472, rs3219489, and rs3219493), thymine DNA glycosylase (TDG) (rs4135054), and single-strand-selective monofunctional uracil–DNA glycosylase 1 (SMUG1) (rs3087404)—was determined using TaqMan^®^ SNP Genotyping Assays and the TaqMan Universal PCR Master Mix, No UNG (Applied Biosystems, Foster City, CA, USA). The total volume of the PCR reaction was 20 μL, including 4 µL of 5× HOT FIREPol^®^ Probe qPCR Mix (Solis, Tartu, Estonia), 1 µL of DNA (100 ng), 1 µL of 20× TaqMan SNP primers, and 14 µL of RNA-free water. PCR reaction conditions were as follows: polymerase activation (10 min, 95 °C), followed by 30 cycles of denaturation (15 s, 95 °C) and annealing/extension (60 s, 60 °C). Genotype was determined using the Bio-Rad CFX96 system (Bio-Rad, Hercules, CA, USA).

The SNPs were selected based on their functional annotation (such as exonic or promoter variants), prior associations with oxidative DNA damage, inflammation, or BER efficiency in published studies, and their minor allele frequency (MAF) in European populations, to ensure adequate statistical power.

### 4.4. Statistical Analysis

A comprehensive statistical analysis of allele and genotype frequencies, along with an assessment of Hardy–Weinberg equilibrium, was conducted using SNPStats online software (https://www.snpstats.net/start.htm, accessed on 14 April 2025) [52]. This analysis examined four distinct genetic models—codominant, dominant, recessive, and overdominant—each offering unique insights into the relationship between genetic variation and multiple sclerosis (MS) risk.

The codominant model, which is the most commonly used, states that each genotype has a separate and independent risk of developing MS. In this model, heterozygous (carrying one variant allele and one common allele) and homozygous genotypes for the variant allele are grouped with homozygous genotypes for the most common allele. This provides a more detailed understanding of how different genotypes impact disease risk. Conversely, the dominant and recessive models suggest that simply having the variant allele can significantly increase the risk of MS. The dominant model indicates that possessing just one variant allele (heterozygous) raises the risk. In contrast, the recessive model shows that only individuals with homozygous genotypes for the variant allele have a higher susceptibility to the disease. The overdominant model adopts a different perspective, proposing that only heterozygous individuals contribute to MS risk, and emphasizing the potential unique role of having one copy of the variant allele.

A chi-squared test was used to thoroughly analyze the differences in genotype distributions between patients with MS and healthy controls. This test is beneficial for determining whether the observed frequency differences are statistically significant. Additionally, a linear regression model was applied to estimate the risk of developing MS, presented as the odds ratio (OR) and a 95% confidence interval (CI). A *p*-value threshold of <0.05 was set to indicate statistical significance, meaning there is less than a 5% chance that the observed differences are due to random variation.

Bonferroni’s correction was applied to control for type I errors from multiple comparisons, adjusting *p*-values to ensure reliable conclusions. A priori power analysis was conducted using QUANTO software (v1.2.4) (https://keck.usc.edu/biostatistics/, accessed on 21 April 2025) [53] to estimate the minimum sample size needed to detect a significant association between selected SNPs and MS risk, with 80% power (β = 0.2) and a two-sided significance level of α = 0.05. The analysis assumed a log-additive genetic model, with disease prevalence set at 0.0013 (based on MS prevalence in Poland) and a minor allele frequency (MAF) range of 0.1 to 0.4, consistent with reported frequencies for the selected SNPs in European populations. Calculations were based on an odds ratio (OR) of 1.8 for risk alleles, reflecting a moderate genetic effect size common in candidate gene studies. These parameters indicated a minimum of about 95–110 cases and controls to achieve adequate power. The final cohort (102 patients with MS and 118 healthy controls) met these requirements. This method complies with the guidelines in [53] described for practical sample size estimation in genetic association studies.

Epistatic interactions were tested by including pairwise interaction terms between biologically plausible SNP pairs in the logistic regression model (e.g., OGG1–XRCC1, SMUG1–TDG). Selection was based on shared involvement in the BER pathway and prior association signals.

### 4.5. Linkage Disequilibrium and Association Analyses

Pairwise linkage disequilibrium (LD) between the genotyped SNPs was evaluated using the squared correlation coefficient (r^2^), calculated in R based on additive encoding of genotypes (0/1/2). SNPs within the same gene that exhibited high LD (r^2^ ≥ 0.8) were analyzed as haplotypes using the haplo.stats package. Haplotype frequencies were estimated with the expectation–maximization (EM) algorithm (haplo.em), and associations with disease status were tested using the score-based method (haplo.score) with additive effect modeling. Global *p*-values and haplotype-specific statistics are reported.

For the remaining SNPs not in linkage disequilibrium (r^2^ < 0.1), a multivariable logistic regression model was built to assess their combined association with multiple sclerosis. Genotypes were numerically coded assuming additive inheritance, and all SNPs were included simultaneously in the model. Odds ratios (ORs) and 95% confidence intervals (CIs) were reported. No additional correction for multiple testing was applied, as the multivariable model naturally adjusts for the number of predictors. All statistical analyses were conducted in R version 4.51 (R Foundation for Statistical Computing, Vienna, Austria).

## 5. Conclusions

Our study suggests that genetic variations within BER genes may be associated with MS. Based on these findings, future research should aim to confirm these associations in larger, more diverse populations and investigate gene–environment interactions that could influence disease risk. Functional assays are necessary to evaluate the impact of the identified SNPs on DNA repair, gene expression, and protein function. Our recent study, which combined comet assay kinetics, BER gene expression, and SNP analysis, showed that patients with MS have a reduced DNA repair capacity that is associated with specific BER-related variants, including rs3087404 and rs4135054 [54]. Integrating genomic data with quantitative MRI measures—such as lesion burden, brain atrophy, and markers of chronic active lesions—may improve the understanding of genotype–phenotype relationships. Ultimately, these multimodal approaches could help identify biomarkers for MS prognosis, treatment response, or personalized risk assessment. However, our study has limitations—the pilot study results need to be validated in a larger sample. Further research is needed to investigate the role of BER gene SNPs in the development of MS in the Polish population.

## Figures and Tables

**Table 1:** Demographic and clinical characteristics of patients with MS.

Variable	Patients with MS (*n* = 102)
Age (Mean ± SD)	42.2 (±13.12)
Female	42.29 (±13.02)
Male	42.05 (±13.43)
Sex	
Female	62
Male	40
MS Type	
Relapsing–Remitting (RRMS)	98
Primary Progressive (PPMS)	3
Secondary Progressive (SPMS)	1
EDSS Score (Mean ± SD)	3.44 (±1.87)

**Table 2:** The Hardy–Weinberg equilibrium analysis *.

Polymorphism/Gene	*p*-Value		
	Totality	Control Group	Study Group
rs25478/*XRCC1*	<0.0001	0.22	<0.0001
rs1052133/*hOGG1*	0.024	0.079	0.16
rs246079/*UNG*	0.5	1	0.24
rs151095402/*UNG*	2.00 × 10^−4^	0.025	0.0025
rs2307293/*MBD4*	0.14	1	0.015
rs3219472/*MUTYH*	0.81	0.36	0.45
rs3219489/*MUTYH*	0.64	0.16	0.45
rs3219493/*MUTYH*	0.00089	0.19	0.0018
rs4135054/*TDG*	0.023	0.071	1
rs3087404/*SMUG1*	0.027	0.071	0.32

* A Bonferroni correction for multiple comparisons was applied, considering the analysis of 10 SNPs. The adjusted significance threshold was *p* < 0.005 (α = 0.05/10). *p*-values below this threshold were considered statistically significant after correction.

**Table 3:** Basic information and allele frequencies of the 10 selected SNPs.

SNP (Gene Name)	Chr *	Positions **	Allele	Minor Allele Frequency	
				Case	Control
rs25478 (*XRCC1*)	19	43545709	T/T	0.09	0.01
rs1052133 (*OGG1*)	3	9757089	G/G	0.06	0.1
rs246079 (*UNG*)	12	109109255	G/G	0.27	0.22
rs151095402 (*UNG*)	12	109098561	T/T	0.02	0.02
rs2307293 (*MBD4*)	3	129431542	T/T	0.01	0.01
rs3219472 (*MUTYH*)	1	45338378	T/T	0.03	0.02
rs3219489 (*MUTYH*)	1	45331833	G/G	0.03	0.02
rs3219493 (*MUTYH*)	1	45330597	G/G	0.06	0.02
rs4135054 (*TDG*)	12	103969832	T/T	0.02	0.17
rs3087404 (*SMUG1*)	12	54187830	C/C	0.09	0.03

* Chromosome; ** chromosome position according to the Genome Reference Consortium Human Build 38.

**Table 4:** Correlation of MS with the frequency of genotypes of the BER genes.

Polymorphism/Gene	Model	Genotype	Control Group	Study Group	OR (95% CI)	*p*-Value
rs25478 (*XRCC1*)	Codominant	G/G	108 (91.5%)	87 (85.3%)	1.00	0.011
		G/T	9 (7.6%)	6 (5.9%)	0.83 (0.28–2.41)	
		T/T	1 (0.8%)	9 (8.8%)	**11.17 (1.39–89.88)**	
	Dominant	G/G	108 (91.5%)	87 (85.3%)	1.00	0.15
		G/T-T/T	10 (8.5%)	15 (14.7%)	1.86 (0.80–4.35)	
	Recessive	G/G-G/T	117 (99.2%)	93 (91.2%)	1.00	0.0028
		T/T	1 (0.8%)	9 (8.8%)	**11.32 (1.41–90.97)**	
	Overdominant	G/G-T/T	109 (92.4%)	96 (94.1%)	1.00	0.61
		G/T	9 (7.6%)	6 (5.9%)	0.76 (0.26–2.20)	
rs1052133 (*OGG1*)	Codominant	C/C	41 (34.8%)	46 (45.1%)	1.00	0.21
		C/G	65 (55.1%)	50 (49%)	0.69 (0.39–1.20)	
		G/G	12 (10.2%)	6 (5.9%)	0.45 (0.15–1.29)	
	Dominant	C/C	41 (34.8%)	46 (45.1%)	1.00	0.12
		C/G-G/G	77 (65.2%)	56 (54.9%)	0.65 (0.38–1.12)	
	Recessive	C/C-C/G	106 (89.8%)	96 (94.1%)	1.00	0.24
		G/G	12 (10.2%)	6 (5.9%)	0.55 (0.20–1.53)	
	Overdominant	C/C-G/G	53 (44.9%)	52 (51%)	1.00	0.37
		C/G	65 (55.1%)	50 (49%)	0.78 (0.46–1.33)	
rs246079 (*UNG*)	Codominant	A/A	32 (27.1%)	29 (28.4%)	1.00	0.55
		A/G	60 (50.9%)	45 (44.1%)	0.83 (0.44–1.56)	
		G/G	26 (22%)	28 (27.4%)	1.19 (0.57–2.47)	
	Dominant	A/A	32 (27.1%)	29 (28.4%)	1.00	0.83
		A/G-G/G	86 (72.9%)	73 (71.6%)	0.94 (0.52–1.69)	
	Recessive	A/A-A/G	92 (78%)	74 (72.5%)	1.00	0.35
		G/G	26 (22%)	28 (27.4%)	1.34 (0.72–2.48)	
	Overdominant	A/G	58 (49.1%)	57 (55.9%)	0.76	0.32
		A/A-G/G	60 (50.9%)	45 (44.1%)	1.00 (0.45–1.30)	
rs151095402 (*UNG*)	Codominant	C/C	108 (91.5%)	97 (95.1%)	1.00	0.41
		C/T	8 (6.8%)	3 (2.9%)	0.42 (0.11–1.62)	
		T/T	2 (1.7%)	2 (2%)	1.11 (0.15–8.06)	
	Dominant	C/C	108 (91.5%)	97 (95.1%)	1.00	0.29
		C/T-T/T	10 (8.5%)	5 (4.9%)	0.56 (0.18–1.69)	
	Recessive	C/C-C/T	116 (98.3%)	100 (98%)	1.00	0.88
		T/T	2 (1.7%)	2 (2%)	1.16 (0.16–8.39)	
	Overdominant	C/C-T/T	110 (93.2%)	99 (97.1%)	1.00	0.18
		C/T	8 (6.8%)	3 (2.9%)	0.42 (0.11–1.61)	
rs2307293 (*MBD4*)	Codominant	C/C	97 (82.2%)	100 (98%)	1.00	<0.0001
		C/T	20 (16.9%)	1 (1%)	**0.05 (0.01–0.37)**	
		T/T	1 (0.8%)	1 (1%)	0.97 (0.06–15.73)	
	Dominant	C/C	97 (82.2%)	100 (98%)	1.00	<0.0001
		C/T-T/T	21 (17.8%)	2 (2%)	**0.09 (0.02–0.40)**	
	Recessive	C/C-C/T	117 (99.2%)	101 (99%)	1.00	0.92
		T/T	1 (0.8%)	1 (1%)	1.16 (0.07–18.76)	
	Overdominant	C/C-T/T	98 (83%)	101 (99%)	1.00	<0.0001
		C/T	20 (16.9%)	1 (1%)	**0.05 (0.01–0.37)**	
rs3219472 (*MUTYH*)	Codominant	C/C	77 (65.2%)	75 (73.5%)	1.00	0.26
		C/T	39 (33%)	24 (23.5%)	0.63 (0.35–1.15)	
		T/T	2 (1.7%)	3 (2.9%)	1.54 (0.25–9.48)	
	Dominant	C/C	77 (65.2%)	75 (73.5%)	1.00	0.18
		C/T-T/T	41 (34.8%)	27 (26.5%)	0.68 (0.38–1.21)	
	Recessive	C/C-C/T	116 (98.3%)	99 (97.1%)	1.00	0.54
		T/T	2 (1.7%)	3 (2.9%)	1.76 (0.29–10.73)	
	Overdominant	C/C-T/T	79 (67%)	78 (76.5%)	1.00	0.12
		C/T	39 (33%)	24 (23.5%)	0.62 (0.34–1.13)	
rs3219489 (*MUTYH*)	Codominant	C/C	73 (61.9%)	75 (73.5%)	1.00	0.04
		C/G	43 (36.4%)	24 (23.5%)	**0.54 (0.30–0.98)**	
		G/G	2 (1.7%)	3 (2.9%)	1.46 (0.24–8.99)	
	Dominant	C/C	73 (61.9%)	75 (73.5%)	1.00	0.065
		C/G-G/G	45 (38.1%)	27 (26.5%)	0.58 (0.33–1.04)	
	Recessive	C/C-C/G	116 (98.3%)	99 (97.1%)	1.00	0.54
		G/G	2 (1.7%)	3 (2.9%)	1.76 (0.29–10.73)	
	Overdominant	C/C-G/G	75 (63.6%)	78 (76.5%)	1.00	0.037
		C/G	43 (36.4%)	24 (23.5%)	**0.54 (0.30–0.97)**	
rs3219493 (*MUTYH*)	Codominant	C/C	100 (84.8%)	81 (79.4%)	1.00	0.23
		C/G	16 (13.6%)	15 (14.7%)	1.16 (0.54–2.48)	
		G/G	2 (1.7%)	6 (5.9%)	3.70 (0.73–18.85)	
	Dominant	C/C	100 (84.8%)	81 (79.4%)	1.00	0.3
		C/G-G/G	18 (15.2%)	21 (20.6%)	1.44 (0.72–2.88)	
	Recessive	C/C-C/G	116 (98.3%)	96 (94.1%)	1.00	0.093
		G/G	2 (1.7%)	6 (5.9%)	3.62 (0.72–18.37)	
	Overdominant	C/C-G/G	102 (86.4%)	87 (85.3%)	1.00	0.81
		C/G	16 (13.6%)	15 (14.7%)	1.10 (0.51–2.35)	
rs4135054 (*TDG*)	Codominant	C/C	53 (44.9%)	73 (71.6%)	1.00	<0.0001
		C/T	45 (38.1%)	27 (26.5%)	**0.44 (0.24–0.79)**	
		T/T	20 (16.9%)	2 (2%)	**0.07 (0.02–0.32)**	
	Dominant	C/C	53 (44.9%)	73 (71.6%)	1.00	1 × 10^−4^
		C/T-T/T	65 (55.1%)	29 (28.4%)	**0.32 (0.18–0.57)**	
	Recessive	C/C-C/T	98 (83%)	100 (98%)	1.00	1 × 10^−4^
		T/T	20 (16.9%)	2 (2%)	**0.10 (0.02–0.43)**	
	Overdominant	C/C-T/T	73 (61.9%)	75 (73.5%)	1.00	0.065
		C/T	45 (38.1%)	27 (26.5%)	0.58 (0.33–1.04)	
rs3087404 (*SMUG1*)	Codominant	T/T	93 (78.8%)	57 (55.9%)	1.00	0.0012
		T/C	21 (17.8%)	36 (35.3%)	**2.80 (1.49–5.26)**	
		C/C	4 (3.4%)	9 (8.8%)	**3.67 (1.08–12.47)**	
	Dominant	T/T	93 (78.8%)	57 (55.9%)	1.00	3 × 10^−4^
		T/C-C/C	25 (21.2%)	45 (44.1%)	**2.94 (1.63–5.30)**	
	Recessive	T/T-T/C	114 (96.6%)	93 (91.2%)	1.00	0.086
		C/C	4 (3.4%)	9 (8.8%)	2.76 (0.82–9.24)	
	Overdominant	T/T-C/C	97 (82.2%)	66 (64.7%)	1.00	0.0031
		T/C	21 (17.8%)	36 (35.3%)	**2.52 (1.35–4.70)**	

**Table 5:** Haplotype analysis of MUTYH variants (rs3219489 and rs3219472).

Haplotype	Frequency	Score	*p*-Value
C–C	0.82270	1.59113	0.1116
C–T	0.00230	-	-
G–C	0.01139	−2.10388	0.0354
G–T	0.16361	−0.88701	0.3751

**Table 6:** Multilocus logistic regression for non-MUTYH SNPs.

SNP	OR	95% CI (Lower)	95% CI (Upper)	*p*-Value
rs25478 (*XRCC1*)	2.00	0.99	4.51	0.068
rs1052133 (*OGG1*)	0.57	0.33	0.97	0.043
rs246079 (*APE1*)	0.83	0.53	1.27	0.383
rs3087404 (*SMUG1*)	1.98	1.17	3.46	0.013
rs151095402 (*UNG*)	0.65	0.25	1.52	0.332
rs2307293 (*MBD4*)	0.16	0.03	0.54	0.010
rs4135054 (*TDG*)	0.38	0.23	0.61	0.0001
rs3219493 (*MUTYH*)	0.60	0.29	1.18	0.150

## Data Availability

The data presented in this study are available on request from the corresponding author.

## Abbreviations

MS: multiple sclerosis; CNS: central nervous system; RRMS: relapsing–remitting multiple sclerosis; PPMS: primary progressive multiple sclerosis; SPMS: secondary progressive multiple sclerosis; DMT: disease-modifying therapy; EDSS: Expanded Disability Status Scale; MRI: magnetic resonance imaging; ROS: reactive oxygen species; GWAS: genome-wide association study; SNP: single-nucleotide polymorphism; SNPs: single-nucleotide polymorphisms; BER: base excision repair; DNA: deoxyribonucleic acid; OR: odds ratio; CI: confidence interval; HWE: Hardy–Weinberg equilibrium; LD: linkage disequilibrium; MAF: minor allele frequency; PCR: polymerase chain reaction; TE buffer: Tris/EDTA buffer; APE1: apurinic/apyrimidinic endonuclease 1; hOGG1: human 8-oxoguanine DNA glycosylase; hNTH1: human endonuclease III-like protein 1; hNEIL1/2: human endonuclease VIII-like proteins 1 and 2; hMYH: human MutY homolog; SMUG1: single-strand-selective monofunctional uracil–DNA glycosylase; TDG: thymine DNA glycosylase; XRCC1: X-ray repair cross-complementing protein 1; MBD4: methyl-CpG-binding domain protein 4; MUTYH: MutY DNA glycosylase; UNG: uracil–DNA glycosylase.
